# Wearable Ultrasound-Imaging-Based Visual Feedback (UVF) Training for Ankle Rehabilitation of Chronic Stroke Survivors: A Proof-of-Concept Randomized Crossover Study

**DOI:** 10.3390/bios15060365

**Published:** 2025-06-06

**Authors:** Yu-Yan Luo, Chen Huang, Zhen Song, Vaheh Nazari, Arnold Yu-Lok Wong, Lin Yang, Mingjie Dong, Mingming Zhang, Yong-Ping Zheng, Amy Siu-Ngor Fu, Christina Zong-Hao Ma

**Affiliations:** 1 Department of Biomedical Engineering, The Hong Kong Polytechnic University, Hong Kong SAR, China; yuyan-laura.luo@connect.polyu.hk (Y.-Y.L.); cece.huang@connect.polyu.hk (C.H.); zhen0212.song@connect.polyu.hk (Z.S.); v.nazari@uq.edu.au (V.N.); yongping.zheng@polyu.edu.hk (Y.-P.Z.); 2 Research Institute for Smart Ageing, The Hong Kong Polytechnic University, Hong Kong SAR, China; arnold.wong@polyu.edu.hk; 3 Department of Rehabilitation Sciences, The Hong Kong Polytechnic University, Hong Kong SAR, China; amy.fu@polyu.edu.hk; 4 School of Nursing, The Hong Kong Polytechnic University, Hong Kong SAR, China; l.yang@polyu.edu.hk; 5 Faculty of Materials and Manufacturing, Beijing University of Technology, Beijing 100124, China; dongmj@bjut.edu.cn; 6 Department of Biomedical Engineering, Southern University of Science and Technology, Shenzhen 518000, China; zhangmm@sustech.edu.cn

**Keywords:** muscle training, stroke, tibialis anterior, ultrasound imaging, visual feedback

## Abstract

This study investigated the effect of wearable ultrasound-imaging-based visual feedback (UVF) on assisting paretic ankle dorsiflexion training of chronic stroke survivors. Thirty-three participants with unilateral hemiplegia performed maximal isometric contractions on an isokinetic dynamometer in randomized conditions with and without UVF that provided by a wearable ultrasound imaging system. Torque parameters (mean, peak, percentage of maximal voluntary contraction) and tibialis anterior muscle thickness were analyzed across different contraction phases. Statistical comparisons were conducted using paired *t*-tests or Wilcoxon tests. Correlation analyses were performed using Pearson’s or Spearman’s tests. Results demonstrated that UVF significantly improved torque output, as evidence by the increased percentage of maximal voluntary contraction (%MVC) during entire contractions (*p* = 0.007), increased mean (*p* ≤ 0.022) and peak (*p* ≤ 0.044) torque and the %MVC (*p* ≤ 0.004) during mid and end phases, and larger muscle thickness during mid contraction (*p* = 0.045). Moderate correlations were found between torque and muscle thickness (r ≥ 0.30, *p* ≤ 0.049). These findings preliminarily supported the positive outcomes of real-time wearable UVFs in enhancing paretic ankle dorsiflexion strength and force control during isometric contractions in chronic stroke survivors. While the developed and validated new training protocol may potentially serve as a practical adjunct to existing rehabilitation approaches, further investigations emphasizing the functional outcomes and clinical translations are still needed to verify the clinical utility.

## 1. Introduction

Stroke has been a significant global public health challenge, and is ranked as the second leading cause of morbidity and mortality worldwide [1]. This has warranted the urgent need for developing and validating effective prevention and intervention strategies. In China, the prevalence of stroke has shown a persistent rise in recent years and reached a rate of 2.58% in 2019 [2], with an incidence of 345.1 per 100,000 person-years [3]. Consequently, a growing population of stroke survivors have continuously been suffering from significant consequences, including impaired mobility, cognition, and emotional well-being.

Post-stroke skeletal muscle dysfunction has remained a critical rehabilitation challenge and is primarily driven by two interrelated pathologies: (1) sarcopenia and (2) spasticity. Sarcopenia, which is defined as acute and non-age-dependent muscle wasting caused by neurogenic catabolic signaling from brain lesions [4], affects approximately 33–52% of stroke survivors [5]. The mechanisms include muscle denervation [6], fast-twitch fiber predominance [7], and catabolic/anabolic imbalance [8]. Concurrently, spasticity (defined as velocity-dependent hypertonia) [9] occurs in around 20–40% of patients with stroke [10,11]. It exacerbates motor impairments through the hyperexcitable stretch reflexes [12]. These conditions synergistically impair muscle strength and motor control, leading to functional limitations of deteriorated gait stability and increased fall risk [13].

Notably, paretic limb weakness has been a central barrier to the motor recovery of stroke survivors [14,15]. Previous studies have reported that the ankle dorsiflexor strength of the paretic limb is approximately 40% to 50% weaker than that of the non-paretic side [16,17]. The paretic ankle dorsiflexor weakness has also been identified as a determinant factor of poor walking performance in patients with hemiplegic stroke [18]. Muscle weakness commonly results in a reduced ability to clear the foot over the ground during the swing phase, leading to an altered gait pattern with decreased stability and increased risk of falls [18].

Muscle strengthening, or resistance training, involves performing exercises with resistance against the muscles to counteract the muscles to generate force. It elicits adaptive changes in both muscle and central nervous system function [19]. It can offer a wide range of benefits, including improved muscular strength [20], muscle hypertrophy [21], motor control [22], and overall physical performance [18]. This is why muscle strengthening has been essential in the rehabilitation of stroke survivors. Several systematic reviews have provided strong evidence support for the overall effectiveness of lower limb muscle strength training in enhancing strength and functional ability among stroke survivors [23,24].

Visual feedback training has been extensively researched in stroke rehabilitation. It can empower stroke survivors with improved overall performance by facilitating the real-time visualization of actual performance and active comparison to the expected goals. The reliance on visual feedback tends to increase in stroke survivors [25]. As a result, the visual feedback accelerates the motor learning process and helps sustain motivation during motor learning in stroke survivors [26]. Most previous studies on stroke rehabilitation have provided visual feedback via pre-recorded or real-time visual graphic cues that derive from physiological signals, including muscle electrical activity (i.e., electromyography or EMG) [27], force generation [28,29], and kinematic information [30]. These approaches mainly involve external monitoring of muscle activation and contraction outputs. Meanwhile, previous studies have reported that measuring the internal structure of paretic muscles could provide more information and difference between the paretic and non-paretic limbs than that of the conventional kinetic and EMG measurements in stroke survivors [31,32]. However, changes in muscles’ internal structures during training have remained unclear.

Wearable ultrasound imaging has the advantage of providing direct visualization of the internal structure or morphology of a target skeletal muscle in stroke individuals [31,32], enabling more in-depth non-invasive monitoring and examination of muscle contraction patterns during different activities (or sonomyography (SMG)). The utilization of ultrasound imaging as a means of visual biofeedback for muscle contraction has been explored mainly in muscles that are difficult to control voluntarily. Previous studies have applied ultrasound imaging to feedback the contraction of pelvic floor muscles in pregnant women [33], and deep trunk muscles for individuals with low back pain [34,35,36]. However, its effect on stroke survivors has remained unclear and warrants further investigation.

Ultrasound-imaging-based visual feedback (UVF) could offer an innovative approach to directly monitor paretic muscle contraction patterns during the strengthening training of stroke survivors. It enables the real-time visualization of muscle contraction patterns through ultrasound imaging [37], such that patients can better control the voluntary contraction of targeted/paretic muscles. It is anticipated that UVF could serve as a new rehabilitative solution for augmenting muscle strengthening of stroke survivors. Previous studies on healthy adults have supported the efficacy of UVF in enhancing neuromuscular control. Specifically, UVF has been shown to enhance precision during isometric gastrocnemius contractions [38] and increase the force output in isotonic pectoralis major exercises [39]. These findings suggest its potential for stroke rehabilitation, where sensorimotor and integrative dysfunctions often impair voluntary force generation due to disrupted neuromuscular control [40]. By providing the real-time visualization of muscle contraction patterns, UVF could help address these deficits and enable survivors to regain controlled paretic muscle activation and strengthening. Additionally, UVF may support motor learning by facilitating the activation of specific muscles and restoring functional movement in stroke survivors. However, no prior studies have yet explored its application in stroke rehabilitation.

Thus, this proof-of-concept study aimed to (1) investigate the effects of UVF on ankle dorsiflexion torque and tibialis anterior (TA) muscle thickness during isometric contractions in chronic stroke survivors; and (2) explore the relationship between changes in ankle dorsiflexion torque and TA muscle thickness during contraction. It was hypothesized that UVF would lead to greater increases in ankle dorsiflexion torque and TA muscle thickness during isometric ankle dorsiflexion compared with conditions without UVF. The findings could provide new insights for developing rehabilitation strategies to restore paretic muscle strength and function in stroke survivors.

## 2. Materials and Methods

### 2.1. Study Design

The current proof-of-concept study employed a randomized crossover research design. The sequence of training with UVF and training without UVF was randomly assigned to each stroke participant, using a computer-generated number sequence. The generated random number file was encrypted, securely stored, and kept confidential from all participants. All procedures were performed in accordance with the Consolidated Standards of Reporting Trials (CONSORT) 2010 statement: Extension to Randomized Crossover Trials [41] to ensure comprehensive and transparent reporting of the research process and findings. The CONSORT checklist for reporting the randomized crossover trials was used to enhance the reporting quality (Table S1).

### 2.2. Participants

A total of 33 community-dwelling stroke survivors were recruited through convenience sampling from the local community in Hong Kong, using social media advertisements. Individuals were considered eligible if they fulfilled the following inclusion criteria: (1) having unilateral hemiplegia; (2) being at least 12 months since stroke onset; (3) living in the community; (4) being able to walk independently without assistive devices for at least 10 m; and (5) demonstrating adequate cognitive ability to follow experimental instructions. Individuals were excluded if they had the following: (1) cerebellar or brain stem strokes; (2) any peripheral or central nervous system dysfunction; (3) significant ankle muscle spasticity as indicated by a modified Ashworth scale score greater than 1+; or (4) unstable medical conditions (i.e., uncontrolled hypertension, arrhythmias, heart disorders, or other diagnosed serious illnesses).

The sample size was determined using G*Power 3.1 (Erdfelder, Faul, & Buchner, 1996). Based on the preliminary results from the pilot study on six participants, an effect size of 0.510 was calculated. The mean differences and standard deviations of dorsiflexion torque measurements were compared between UVF and non-UVF conditions to calculate the effect size. With the conventional standards of a 0.05 significance level and 80% statistical power, the statistical analysis showed that 33 participants were needed to reliably detect meaningful differences in this study. The actual statistical power of 0.811 based on 33 participants also doubly confirmed the appropriate estimation of the sample size in the current study.

This study was granted ethical approval by the Institutional Review Board of The Hong Kong Polytechnic University (Reference No.: HSEARS20230801001; Date: 12 October 2023). Written informed consent was obtained from all participants before the start of this study. This study was also registered in the World Health Organization International Clinical Trials Registry Platform (ICTRP) via the Chinese Clinical Trial Registry (ChiCTR, Reference No.: ChiCTR2300073454; Date: 11 July 2023; Website: https://www.chictr.org.cn/showprojEN.html?proj=200938).

### 2.3. Equipment

A wearable ultrasound imaging system (defined as “wearable SMG system” in this study) was used to allow participants to visualize their internal muscle contraction patterns in real-time during training, and to capture the muscle thickness changes throughout the experiment (imaging width: 38.4 mm, depth: 60 mm). The system consisted of a customized wireless linear ultrasound probe (Bandwidth 7.5 MHz ± 35%; frame rate: 20 Hz) that connected to a laptop via Wi-Fi communication. All the ultrasound images were captured using the same wearable SMG system (frequency: 20 Hz) in B-mode, with a depth of 60 mm, a dynamic range of 40 dB, and a pre-set averaged gain of +40 dB.

The test-retest and inter-rater reliability of the mobile SMG system was examined [42] using the measured tibialis anterior (TA) muscle thickness data from three participants while performing maximal isometric voluntary contraction (MIVC). Specifically, the test-retest reliability was evaluated using (1) resting TA muscle thickness (captured continuously for one second at 20 Hz preceding MIVC initiation, yielding 20 discrete measurement points) and (2) maximum TA muscle thickness during MIVC from two reproducible trials (i.e., trials with less than 10% variability). The inter-rater reliability was assessed for the measurements from two independent researchers, who twice measured the TA muscle thickness on all ultrasound images following the standardized protocols. The applied wearable SMG system demonstrated excellent inter-rater (ICC ≥ 0.997) and test-retest (ICC ≥ 0.993) reliability in both analyses, supporting its measurement consistency for this study (Table S2).

### 2.4. Protocol of Providing Ultrasound-Imaging-Based Visual Feedback (UVF)

An experienced researcher (with over eight years of experience in medical ultrasound imaging) performed and monitored the placement of the wearable ultrasound probe throughout the experiment for all participants. The ultrasound probe was placed longitudinally on the muscle belly at the proximal 30% of the line connecting the fibular head and the medial malleolus, with measurements taken in the sitting position [43]. To minimize artifacts and optimize imaging quality, the ultrasound probe was oriented perpendicular to the TA muscle fibers [44]. The gain was systematically stratified during the capture. It was initially set to a baseline of +40 dB, and then fine-tuned layer by layer by an experienced researcher to account for the differential signal attenuation between the superficial fascia, muscle belly, and deep aponeurosis [45]. This adjustment facilitated the simultaneous clarity of all structural layers during the dynamic contractions of TA muscle. An adequate amount of ultrasound gel was applied between the probe and skin surface to maintain optimal imaging quality, before fastening the probe in place using a strap.

A laptop display was placed in front of each participant to provide real-time UVF. The distance between the screen and the participant’s eyes was approximately 1 m. The visual target for TA muscle contraction during the UVF training was determined as the farthest displacement position of the deep aponeurosis that recorded during the MIVC measurement. This position represented the best muscle contraction performance, as determined by the largest muscle thickness in the two repetitions.

### 2.5. Experimental Procedures

Prior to data collection, all participants received a detailed briefing of this study’s objectives and experimental procedures. First, each participant’s demographic information was collected. Their functional outcomes of independence and mobility were evaluated using three validated assessments of the Functional Independence Measure (FIM) [46], the Fugl-Meyer Assessment for Lower Extremity (FMA-LE), and the Short Physical Performance Battery (SPPB) [47].

#### 2.5.1. Setup and Positioning

**Figure 1:**
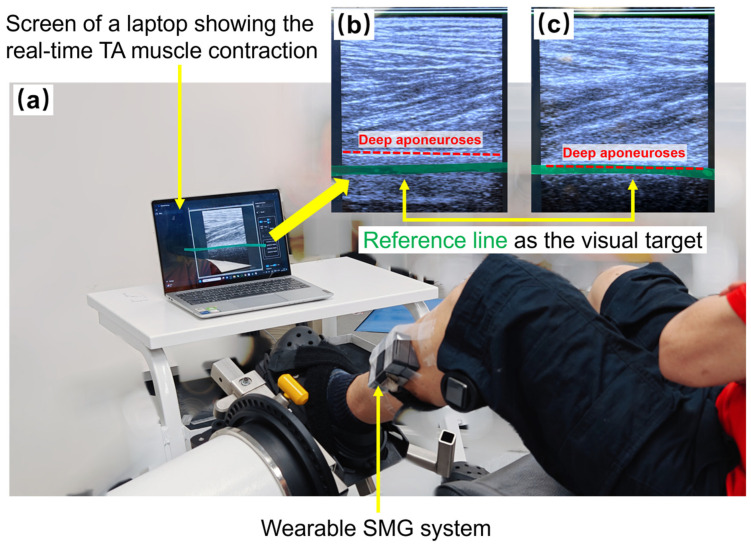
**Experimental setup of ultrasound-imaging-based visual feedback (UVF) muscle training.** (**a**) Placement of wearable ultrasound probe and display for UVF; (**b**) an example of the captured ultrasound image showing the TA muscle during resting phase; (**c**) an example of the captured ultrasound image showing the TA muscle during isometric contraction. **Note:** SMG—sonomyography; TA—tibialis anterior.

Following the baseline assessments, the wearable ultrasound probe was positioned on the TA muscle belly following the protocol as described above. Participants were instructed to sit on an isokinetic dynamometer (CSMi, Stoughton, MA, USA) with the paretic foot securely fastened to the force platform. The ankle joint was fixed in a neutral position, or adjusted to the maximum achievable dorsiflexion if the neural position could not be achieved. The thigh of the testing limb was appropriately supported with hip and knee joints stabilized at 60° flexion [48]. The experimental setup is illustrated in Figure 1. Each participant was reminded again of the safety protocols and procedural requirements before the next procedure.

#### 2.5.2. Warm-Up and Familiarization

Each experimental session commenced with a 5 min warm-up, involving assisted active ankle joint range of motion (ROM) exercises at an angular velocity of 30°/s [49]. Participants were subsequently instructed to identify the TA muscle thickness on the real-time ultrasound image as described above. Each participant was then instructed to perform two submaximal isometric contractions to become familiar with the training task [13,50].

#### 2.5.3. Measurement of MIVC and Maximal TA Thickness

To determine the MIVC and the corresponding maximal TA muscle thickness, each participant was instructed to perform two consecutive contractions with their greatest efforts. If the relative difference in both peak ankle dorsiflexion torque and peak TA muscle thickness between these two MIVCs was within 10%, the two collected MIVCs were considered reproducible, and no further trials were required. The two trials measuring MIVCs were then considered as the two MIVC measurement trials in this study. Conversely, if the difference exceeded 10%, additional trials were conducted until two MIVC measurement trials were achieved [51].

The largest torque from the two MIVC measurement trials was recorded as the MIVC torque (MIVC_Tq_). The maximal muscle thickness value (MIVC_MT_), along with the corresponding position of the deep aponeurosis from the two MIVC measurement trials, was confirmed by the experienced researcher and recorded as the target of ultrasound-based visual feedback for each participant. A reference line of the identified position of the deep aponeurosis was then marked on the laptop display (i.e., predefined visual target), to guide each participant during the UVF trainings.

To examine the intra-subject reproducibility of the identified target on ultrasound images, the intra-class correlation coefficient (ICC) was calculated for the peak TA muscle thickness values of the two MIVC measurement trials from all participants. Excellent intra-subject reproducibility of the identified peak TA muscle thickness was found (ICC = 0.995) (Table S3).

#### 2.5.4. Training Protocol

A single session of MIVC training targeting the maximal paretic TA muscle contraction was then conducted. Participants were instructed to perform MIVC of ankle dorsiflexion under two conditions with randomized sequence: (1) training with UVF, and (2) training without UVF. Each condition involved three repetitions of MIVC of ankle dorsiflexion at the paretic side (i.e., three MIVC training trials for each of the two training conditions). For training with the UVF condition, each participant was instructed to perform ankle dorsiflexion with their greatest efforts, to have the deep aponeurosis of TA muscle reaching the marked reference line on the laptop display. For training without the UVF condition, each participant was instructed to perform ankle dorsiflexion with their greatest efforts only, without any visual cues.

Each MIVC training trial was followed by a one-minute rest interval to minimize the effect of fatigue in participants. To mitigate the potential learning effects, each participant had a five-minute washout time period between the two training conditions [52]. Additional rest time was provided upon the participant’s request.

### 2.6. Outcome Measurements

#### 2.6.1. Ankle Dorsiflexion Torque

The primary outcome of this study was the paretic ankle dorsiflexion torque, as recorded using an isokinetic dynamometer (CSMi, Stoughton, MA, USA) with a sampling frequency of 100 Hz. The torque values were recorded during the two MIVC measurement trials and the two MIVC training conditions with and without UVF.

#### 2.6.2. TA Muscle Thickness

**Figure 2:**
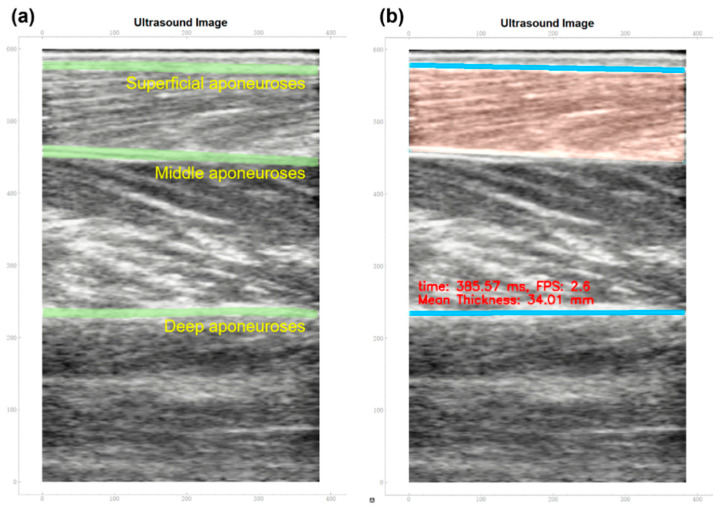
An example of (**a**) the manually tracked aponeuroses of the TA muscle and (**b**) the calculated mean thickness of the muscle in the same frame, as determined by the customized software. **Note:** TA—tibialis anterior.

As shown in Figure 2, the secondary outcome was the difference in paretic TA muscle thickness between the two experimental conditions of training with and without UVF. The ultrasound images of TA muscle contraction of each MIVC trial were measured using the same mobile SMG system for UVF training, and were recorded in a separate video clip. A total of eight video clips were recorded for each participant, consisting of two MIVC measurement trials and six MIVC training trials. Each video clip recorded the resting, MIVC, and re-resting phases of the TA muscle during one MIVC.

TA muscle thickness was measured on each frame of the ultrasound video clips recording TA muscle contraction, using a self-developed SMG software (version 1.0) that was developed based on a customized synthesizing pipeline. The software has been validated in a previous study, with high reliability in extracting and measuring the muscle morphological characteristics (R^2^ > 0.900) [53]. The superficial, middle, and deep aponeuroses of the TA muscle were manually labeled on the first frame of the video clip using the software (Figure 2a).

After labeling and tracking the changes in the superficial, middle, and deep aponeuroses, the TA muscle thickness for each frame of the entire MIVC video clip was automatically measured and calculated by the SMG software using the following formula:

(1) $$MT\left(i\right)=\frac{1}{\left|\omega \right|}\sum_{x\in \omega }m_{i}\left(x\right)-n_{i}(x)$$

where $m_{i}\left(x\right)$ and $n_{i}(x)$ refer to the fitted lines of the superficial aponeurosis and deep aponeurosis in the *i*-th frame of the video, and ω represents the manually defined region of interest from the image.

All the captured ultrasound images were analyzed and labeled by the same experienced researcher (with over eight years of expertise in medical ultrasound imaging). When necessary, the algorithm’s automated myofascial boundary identification function could and would be manually overwritten by the same researcher to maintain measurement accuracy and reliability.

### 2.7. Data Analysis

#### 2.7.1. Ankle Dorsiflexion Torque

The recorded ankle dorsiflexion torque was normalized by dividing it by the body weight of each participant, and was expressed in the unit of Nm/kg. The mean, peak, and coefficient of variance of the normalized ankle dorsiflexion torque for each trial were calculated. The percentage of the ankle dorsiflexion torque relative to the MIVC (%MIVC_Tq_) was calculated using the following equation:

(2) $${\%MIVC}_{Tq}\left(i\right)=\frac{{Torque}_{i}}{{MIVC}_{Tq}}\times 100\%$$

where ${Torque}_{i}$ refers to the recorded absolute ankle dorsiflexion torque at the *i*-th time point (each time point = 0.01 s), and ${MIVC}_{Tq}$ refers to the maximum absolute torque recorded in the two reproducible MIVC measurement trials.

The %MIVC_Tq_ measured participants’ relative torque level in comparison with their maximum torque/strength capacity. Finally, the mean, peak, and coefficient of variation of the normalized ankle torque, along with the mean %MIVC_Tq_ of the absolute ankle dorsiflexion torque for each MIVC training trial, were extracted. These results were further divided into three phases: (1) initial phase (first 1/3 period of the entire contraction), (2) mid phase (middle 1/3 period of the entire contraction), and (3) end phase (last 1/3 period of the entire contraction). All the parameters were analyzed for the entire MIVC contraction and during each of the three phases of contraction.

#### 2.7.2. TA Muscle Thickness

The absolute value of the TA muscle thickness that measured from each frame of the video clip was used for data analysis. Similar to the ankle dorsiflexion torque, the percentage of the MIVC thickness (%MIVC_MT_) was also calculated using the following equation:

(3) $${\%MIVC}_{MT}\left(i\right)=\frac{{TA\;thickness}_{i}}{{MIVC}_{MT}}\times 100\%$$

where ${TA\;thickness}_{i}$ refers to the recorded TA thickness from the *i*-th frame, and ${MIVC}_{MT}$ refers to the maximum thickness recorded in the MIVC measurement trials.

Finally, the mean, peak, coefficient of variation, and the mean %MIVC_MT_ for each MIVC training trial were calculated. Similar to the ankle dorsiflexion torque process, all the TA muscle thickness parameters were analyzed for the entire MIVC contraction and during each of the three phases of the contraction (i.e., initial, mid, and end phases).

### 2.8. Statistical Analysis

Statistical analyses were performed using R Statistical Software (version 4.3.0, R Core Team, 2023). The normality of all parameters was assessed using histogram and Q-Q plot. Based on the data normality, the effects of UVF on the ankle dorsiflexion torque and the TA muscle thickness were examined using the Wilcoxon signed rank tests and paired-*t* tests, respectively. The relationship between the normalized ankle dorsiflexion torque and the absolute TA muscle thickness during contraction was assessed using Pearson’s correlation coefficient for the normally distributed data, and using Spearman’s correlation coefficient for the non-normally distributed data. The significant level for the *p*-value was set at 0.05.

## 3. Results

### 3.1. Pariticipants

**Figure 3:**
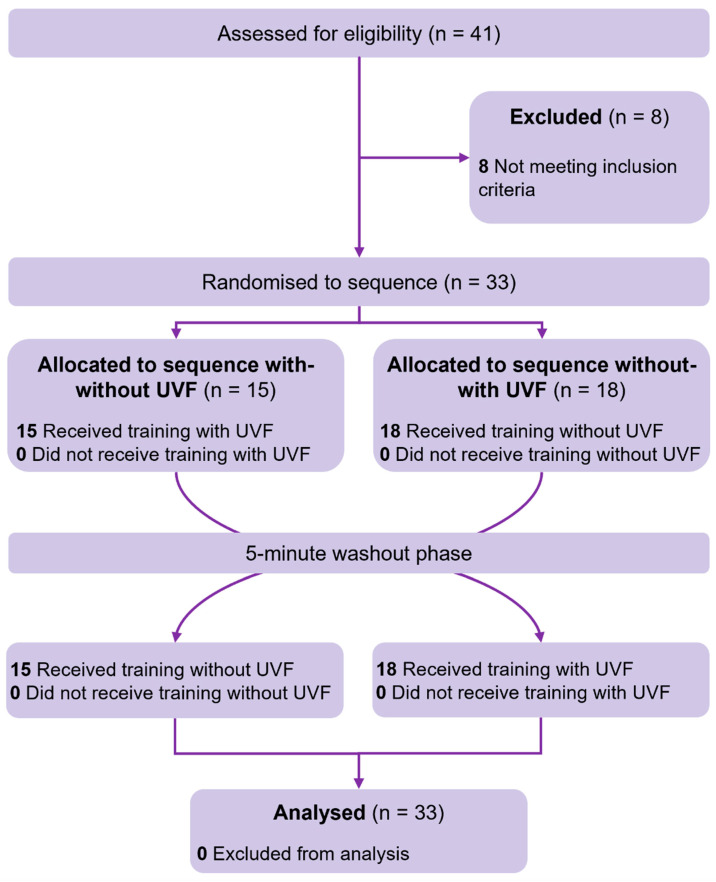
CONSORT flow diagram for crossover trial. **Note:** UVF—ultrasound-imaging-based visual feedback.

As shown in Table 1 and Figure 3, a total of 33 eligible participants (age: 60.5 ± 9.3 years; gender: 39.4% female; BMI: 23.7 ± 2.7 kg/m^2^) participated in this study and were included in the analysis. There were 18 cases of ischemic stroke and 15 cases of hemorrhagic stroke (average post-stroke duration: 6.4 ± 5.5 years). All participants showed a good level of functional independence in performing various activities of daily living, with an average FIM score of 90 (out of 126). They also had relatively good mobility function, based on the FMA-LE and SPPB (sub-)scores on motor function and range of motion of the ankle joint [47].

### 3.2. Effect of UVF on Ankle Dorsiflexion Torque

The overall effects of UVF on ankle dorsiflexion torque are presented in Table 2. The %MIVC_Tq_ of the entire training condition with UVF was 26% significantly larger than that of training without UVF (*p* = 0.007), indicating that contractions with UVF generated an output closer to that of MIVC. While the normalized mean torque was nearly 27% larger in the UVF training condition than that without UVF, there was no statistically significant difference between the two training conditions (*p* = 0.081). Similarly, no significant difference was observed between the two training conditions for the normalized peak torque (*p* = 0.075).

For the three phases of contraction, the normalized peak torque (mid phase: *p* = 0.044; end phrase: *p* = 0.006), normalized mean torque (mid phase: *p* = 0.006; end phrase: *p* = 0.022), and %MIVC_Tq_ (mid phase: 87.9% vs. 72.2%, *p* = 0.001; end phrase: 68.1% vs. 58.6%, *p* = 0.004) of mid and end phases were significantly larger when contracting with UVF than those without UVF. The coefficient of variation during the initial phase was significantly larger for training with UVF than training without UVF (33.1% vs. 30.8%, *p* = 0.007). Conversely, the coefficient of variation during the mid-phase was significantly smaller for training with UVF than training without UVF (9.1% vs. 15.0%, *p* = 0.006). There was no significant difference between the two training conditions during the initial phase for the rest parameters.

### 3.3. Effect of UVF on TA Muscle Thickness

Table 3 summarizes the difference in TA muscle thickness between the two MIVC training conditions with and without UVF. During the mid phase, the peak TA muscle thickness was significantly larger when training with UVF than without (*p* = 0.045), but not for the mean thickness (*p* = 0.092) or the %MIVC_MT_ (*p* = 0.095). The coefficient of variation was also significantly larger when training with UVF during the mid phase than without UVF (*p* = 0.044). No other significant difference in TA muscle thickness was found between the two training conditions.

### 3.4. Relationship Between Ankle Dorsiflexion Torque and TA Muscle Thickness

As shown in Table 4, a moderate positive correlation was observed between the mean normalized ankle dorsiflexion torque and the mean absolute TA muscle thickness during the entire contraction when combining the two training conditions (r = 0.30, *p* = 0.016). Furthermore, moderate positive correlations were found during both the initial (r = 0.37, *p* = 0.003) and the mid (r = 0.30, *p* = 0.013) phases. When the two training conditions were separately analyzed, a moderate positive correlation was only observed during the initial phase of training without UVF in participants (r = 0.36, *p* = 0.040).

As illustrated in Table 5, moderate positive correlations were found between the peak normalized ankle dorsiflexion torque and the peak absolute TA muscle thickness during the entire contraction (r = 0.30, *p* = 0.014), the initial phase (r = 0.35, *p* = 0.005), and the mid phase (r = 0.31, *p* = 0.011), when combining the two training conditions. When analyzed separately, moderate positive correlations were found between the peak normalized ankle dorsiflexion torque and the peak absolute TA muscle thickness during the initial phase of both training conditions with (r = 0.36, *p* = 0.039) and without (r = 0.35, *p* = 0.049) UVF, and during the mid phase of training without UVF (r = 0.36, *p* = 0.043). No additional significant correlations were found.

## 4. Discussion

The present study explored the potential benefits of wearable ultrasound-imaging-based visual feedback (UVF) in strengthening paretic ankle dorsiflexion in community-dwelling stroke survivors. The findings generally supported that utilization of the UVF strategy could generate a larger overall ankle dorsiflexion torque output in stroke participants during isometric muscle strength training. This potentially sheds new light on future stroke rehabilitation research in terms of improving paretic muscle weakness and motor function through the incorporation of UVF, and explores the potential benefits in future clinical practice. However, it is important to note that stroke rehabilitation involves multifaceted challenges, including spasticity and impaired coordination, which may significantly impact the functional outcomes in patients. Further translational research is needed to provide more evidence on how to balance muscle strengthening, spasticity management, and coordination improvement in stroke survivors in future clinical practice.

### 4.1. Effects of UVF on Ankle Dorsiflexion Torque Magnitude and Variation

This study revealed that the developed wearable UVF training strategy could facilitate an overall increased and better controlled force output of paretic ankle dorsiflexion in stroke participants, especially during the mid and end muscle contraction phases. The observed significant improvements in the force parameters, including higher mean and peak ankle dorsiflexion torque, lower coefficient of variation, and higher %MIVC torque, indicate that real-time UVF could improve muscle contraction and enable stroke participants to better regulate their force output by providing a visual stimulus. This augmented ability to achieve and maintain the desired level of force output has enhanced stroke participants’ control over force generation during ankle dorsiflexion. This might be explained by the condition that UVF could allow the stroke participants to have a more precise and straightforward awareness and understanding of their real-time muscle contraction status [54], and to make the necessary adjustments accordingly during the muscle contraction. The current study has observed an overall increased paretic ankle dorsiflexion torque generation, which might be due to an improved voluntary activation of muscle contraction [55] with UVF. This could also be supported by a previous study that has reported a positive correlation between the deficits in voluntary muscle activation and muscle weakness on the paretic side in individuals with chronic stroke [56]. The current study has also found that stroke participants exhibited lower torque variability while training with UVF than without during the mid-phase of contraction. This finding is in accordance with a previous study that reported that, with an augmented visual sensory input, stroke participants and healthy controls had a decreased variability in force output [57]. These findings supported the improved force control during maximum TA muscle contraction, and could be explained by the augmented visual input that activated the visuomotor pathway [58]. Such activation might have influenced the neural signals in motor cortex, either through the projections from the parietal cortex to the premotor cortex [59,60] or from the visual cortex first with the visual stimulus from the real-time UVF [61]. The activation of the parietal cortex has been considered to reflect visuospatial processing [62] and visuomotor error correction [63], which might help explain the findings of the current study. However, the above proposed underlying neural mechanisms should be interpreted with caution, as the current study did not include any neurophysiological measurements to verify. Future research should incorporate techniques such as functional near-infrared spectroscopy (fNIRS) and electroencephalogram (EEG) to obtain direct measurement results. This would help validate the current hypotheses and provide a more complete understanding of UVF’s mechanisms in stroke survivors.

To the best of the authors’ knowledge, the current study has been the first study to apply ultrasound imaging as visual feedback in muscle strengthening training of stroke survivors. Some previous studies have found similar findings in other populations of healthy young adults and patients with chronic pain using a similar UVF strategy with conventional cumbersome wired ultrasound imaging equipment; however, none of them adopted a novel wearable wireless ultrasound probe design. Specifically, a previous study reported that UVF facilitated a closer approximation to the target force intensity, with reduced variability, during gastrocnemius contractions in healthy adults [38]. Another previous study reported an approximate 56% increase in activation levels of the serratus anterior muscle in patients with shoulder pain with UVF training [64]. While most of the previous studies have applied UVF to enhance muscle contraction of healthy adults and patients with shoulder pain, the findings of the current study further supported its effectiveness in clinically improving the paretic ankle muscle weakness of stroke survivors. By enhancing the awareness of muscle activation, stroke individuals could better engage some target muscles. This improved force control could potentially lead to more effective muscle strengthening and motor re-learning for stroke survivors. Future studies could consider exploring the changes in brain activity of stroke survivors to further verify the underlying neurological mechanisms. The long-term effect of UVF on stroke survivors shall also be investigated.

### 4.2. Effects of UVF on TA Muscle Thickness Magnitude and Variation During Contraction

While most ankle dorsiflexion torque parameters improved upon receiving UVF, only the peak TA muscle thickness during the mid contraction phrase significantly improved in stroke participants in this study. Such results partially deviated from the initial hypothesis that a larger TA muscle thickness was expected to be observed in participants during the training condition with UVF. It was intriguing to find that the magnitude of TA muscle thickness during contraction remained relatively consistent among the two training conditions with and without UVF, even though a greater ankle dorsiflexion torque was observed for training with the UVF condition in the current study. This finding was consistent with a previous study, which also reported that, while a significant increase in muscle strength was achieved, the changes in muscle thickness were not statistically significant in healthy individuals following conventional muscle training [65]. Similar findings have also been reported in another previous study, which demonstrated that, while the ankle dorsiflexion torque produced by chronic stroke participants during a MIVC test was significantly lower compared with that of healthy adults, there was no significant difference in TA muscle thickness during contraction between the two subject groups [66]. The non-linear relationship between thickness and activation of the TA muscle may help explain the findings of the current study. Ultrasound imaging has been shown to effectively capture muscle morphological changes during isometric contractions, but primarily at lower intensity levels of under 30% of MIVC [67]. As contraction intensity increased beyond 50% of MIVC, the observed alterations in muscle thickness became relatively minor [67]. This observation has been particularly relevant in the context of the current study, which involved high-intensity contractions at 100% of MIVC. The underlying mechanisms may be explained by the fact that the thickening of the pennately arranged fibers in the TA muscle might be counterbalanced by the changes in fiber angle [68,69,70]. This counterbalance helped keep the surfaces of the origin and insert as parallel and equidistant as possible, which could help maintain a consistent muscle thickness. Previous studies have also reported a significant increase in the pennation angle of the TA muscle from resting to maximum or submaximal MIVC [71,72,73], while no significant changes were observed in TA muscle thickness [72]. Future studies could consider incorporating ultrasound imaging devices with better imaging qualities to verify this. In addition to this, future studies may also consider using the muscle structure of pennation angle as a visual feedback modality, and compare whether the positive paretic muscle strengthening outcome could be further improved to unveil the underlying mechanism.

### 4.3. Relationship Between Ankle Dorsiflexion Torque and TA Muscle Thickness During Contraction

A moderate correlation was found between the normalized ankle dorsiflexion torque and absolute TA muscle thickness during the initial and mid phases of contraction in this study. This correlation was primarily observed when combining the two training conditions and when participants trained without UVF. This might be explained by the previous findings of the counterbalanced changes in Ta muscle fiber angle and thickening of pennately arranged fibers [68,69,70]. It is interesting to observe that, with the addition of UVF, this moderate correlation tended to weaken. This might be because the additional or augmented visual input and stimulus influenced the neuromuscular regulatory output from the motor center, which further altered the existing correlation between muscle contraction and morphological changes. In addition to adopting ultrasound probes with better imaging qualities, future studies could also verify this and unveil the underlying mechanism by evaluating the changes in brain activity of stroke survivors in training conditions with different visual stimuli. Additionally, stroke can lead to various muscle changes in the paretic side, including contractile tissue atrophy, thickening of connective tissue, and alterations in passive mechanical behavior [74]. These pathological changes might further alter the length–force relationship of the impaired muscles [75,76]. Further studies are needed to verify this. The specific relationship between morphological parameters and force output of TA muscle in stroke survivors during different intensities of muscle contractions shall also be further investigated in future studies.

### 4.4. Implications for Future Research and Clinical Practice

The findings of the current study preliminarily underscore the potential of utilizing UVF in the rehabilitative training of paretic ankle dorsiflexion in stroke survivors. The significant improvements in ankle dorsiflexion torque output during isometric muscle strength training supported the potential benefits of UVF in enhancing muscle activation and motor control. However, it shall be noted that this pilot study only demonstrated the positive effect of an isolated muscle, and yet individuals with stroke have multiple joint neuromuscular problems, including undesired flexor tones when performing repetitive dorsiflexion. Future studies can and shall be conducted to further evaluate the effect of UVF on the functional tests of stroke survivors to confirm whether the positive results of the current study can be further transferred to real clinical practice and lead to better rehabilitation outcomes.

It is anticipated that the developed UVF training approach of combining strength and biofeedback training could offer further beneficial effects for stroke survivors in functional performance. Previous studies have reported improvements in ankle muscle strength, balance, and gait performance in stroke survivors who received either ankle biofeedback training [77,78] or ankle muscle strength training [77,78,79], with those receiving feedback showing greater improvements [77,78]. The improved ankle muscle strength and force control after UVF training may help facilitate the effective activation of the TA muscle [80,81] and increase the duration of the single support phase of the paralyzed leg during walking [82]. Additionally, it is anticipated that there may also be improvements in the ankle dorsiflexion angle at heel strike and the toe clearance during the swing phase of a gait cycle [83], as the improved paretic TA muscle strength and force control could help reduce the abnormal foot drop position. These muscle-level improvements may also potentially lead to more noticeable functional enhancements during walking [82], such as improved gait symmetry. This could help improve the stroke survivor’s balance and reduce the risk of falls and associated injuries in daily activities [84]. However, these anticipations should be interpreted with caution, as further studies are needed to verify the potential functional benefits of UVF in stroke survivors.

The observed improved ankle dorsiflexion torque also raised intriguing speculations about the underlying neural mechanisms. Future studies could consider investigating the changes in brain activity that might be associated with UVF training. Both the augmented visual stimulation and voluntary muscle contraction shall be considered in the study design. This may help provide insights on how visual feedback influences the motor control pathways of stroke survivors, and how such information could be considered to refine and improve the future rehabilitative strategy and outcomes for stroke survivors.

The application of a wearable ultrasound imaging system presents an opportunity to expand the evaluated UVF training beyond laboratory settings and into community-based rehabilitation. The flexible mobility and accessibility of this technology could enable stroke survivors to engage in guided feedback-driven strength training in outpatient clinics or even in home environments. This transition may help bridge the gap between isolated muscle training and functional recovery while addressing the practical challenges of expensive cost and high technical complexity. Future implementations should prioritize some user-friendly designs and validate the efficacy of UVF in real-world rehabilitation contexts to promote scalability. In future studies, standardized questionnaires, such as the System Usability Scale (SUS) [85,86] and Post-Study System Usability Questionnaire (PSSUQ) [87,88], should be systematically employed to quantitatively assess user satisfaction and system usability across different clinical and home-based UVF applications. The collected data would be essential to optimize the implementation protocols and patient adherence. Additionally, future studies should investigate the potential rehabilitative benefits of UVF for both survivors of subacute stroke and those with more severe functional impairments (e.g., non-ambulatory patients with minimal ankle dorsiflexion) to establish the intervention’s broader applicability across stroke populations with various levels of physical disabilities.

Moreover, the current study only observed minor changes in TA muscle thickness, despite the increased ankle dorsiflexion torque. These findings highlight not only the complexity of muscle adaptation following stroke but also the limitations of two-dimensional muscle thickness measurements through B-mode ultrasound imaging. The observed changes in muscle structure from the ultrasound imaging may not be able to fully reflect the detailed changes in the muscle activity. Multiple factors may contribute to the observed small changes in muscle thickness, including the narrow width of the ultrasound probe, tightness of the surrounding tissues, and stiff compartment, etc. It is advised that clinicians should not solely rely on the muscle thickness parameter as the indicator of changes in muscle strength or rehabilitation progress. Beyond muscle thickness, previous studies have identified multidimensional structural adaptations in skeletal muscles following stroke, including reduced muscle quality [89] and elevated intramuscular adipose tissue [90]. These changes can also be quantified via ultrasound-derived parameters such as grayscale level [91] and echo intensity [90]. Neuromuscular alterations, such as muscle denervation [6], have been known to contribute to changes in intermuscular tissue composition, which have been associated with muscle weakness [4]. The interaction between these neuromuscular alterations and the muscle structural adaptations underscores the significance of combining electrical signals (e.g., surface electromyography) and ultrasound imaging signals to evaluate the rehabilitation process in the future. In addition to muscle weakness, spasticity and joint stiffness are also major challenges in most patients with chronic stroke in clinical practice. Thus, a comprehensive assessment of muscle function should be prioritized. This shall include a thorough evaluation of morphological changes, neuromuscular control, and functional task performance, with consideration for both short-term and long-term effects. It may also be helpful to investigate the potential relationship between muscle morphological changes and muscle function improvements, to further elucidate how UVF would influence muscle architecture and performance in stroke survivors.

### 4.5. Limitations of This Study

Despite the authors’ best efforts for a robust study design, the present study still held several limitations that could be addressed in future studies. Firstly, this pilot study adopted a randomized crossover design to preliminarily explore the effect of UVF on the paretic ankle dorsiflexion of stroke participants. A randomized controlled trial with at least two groups could be conducted in the future to further verify the reported findings of the developed protocol in stroke survivors.

Secondly, while the immediate changes in TA muscle thickness and torque were thoroughly analyzed, the absence of neurophysiological measurements, such as fNIRS and EMG, limited the identification of the underlying neural mechanisms for this study. Future studies should incorporate these neurophysiological measures to facilitate a more comprehensive understanding of UVF’s mechanisms in lower limb stroke rehabilitation.

Thirdly, while the sitting isometric task minimized compensatory movements, it also precluded the evaluation of functional carryover to gait or other daily activities, particularly given that stroke survivors tend to rely on compensatory strategies of hip/knee flexor co-activation during walking. Future studies should incorporate some functional assessments to evaluate the long-term carryover effects of UVF on daily dynamic tasks, including walking, stair climbing, and sit-to-stand transitions, which could represent more actual real-world mobility challenges.

Fourthly, although the current proof-of-concept study design prioritized a controlled training setup, the repetitive isolated ankle dorsiflexion training may lead to some unintended abnormal consequences. These include exacerbating flexor tone or disrupting inter-joint coordination in dynamic tasks.

Last but not least, this study adopted muscle thickness as the sole UVF parameter. This made it difficult to determine whether some other ultrasound imaging-based parameters or biomarkers could provide better muscle contraction improvement. Future studies could consider verifying whether the other parameters of fascicle length or pennation angle could provide a more comprehensive reflection of changes in muscle architecture and further enhance positive training benefits.

## 5. Conclusions

This study has demonstrated that real-time UVF could significantly enhance the output paretic ankle dorsiflexion force magnitude and control during isometric contractions in community-dwelling stroke survivors. The findings highlight the great potential of UVF as a targeted training tool to address the deficits in isolated ankle dorsiflexion, which has been a critical determinant of functional mobility levels in stroke survivors.

The preliminary positive training outcomes of the introduced UVF protocol support the feasibility of applying it as an engaging adjunct to conventional rehabilitation in future clinical practice. However, future studies shall validate whether these positive outcomes in isolated muscle performance could be translated into functional improvements in daily activities or not. Further identification and optimization of some additional UVF parameters (e.g., fascicle length or pennation angle) and the conduct of some longitudinal trials would also be essential to refine the clinical application and establish some evidence-based protocols for the long-term recovery of individuals with stroke.

## Figures and Tables

**Table 1:** Descriptive information of stroke participants (mean ± SD, *n* = 33).

Item	Value
Gender (female/male)	13/20
Type (ischemic/haemorrhagic)	18/15
Age (year)	60.5 ± 9.3
Height (cm)	164.8 ± 7.8
Weight (kg)	64.3 ± 10.1
BMI (kg/m^2^)	23.7 ± 2.7
Stroke duration (year)	6.4 ± 5.5
FIM	89.0 ± 7.6
FMA-LE motor	21.1 ± 5.8
FMA-ROM DF	0.7 ± 0.8
FMA-ROM PF	1.7 ± 0.6
SPPB	7.2 ± 3.0

**Note:** BMI—body mass index; FIM: Functional Independence Measure; FMA-LE—Fugl-Meyer Lower Extremity motor sub-score; FMA-ROM DF—Fugl-Meyer Lower Extremity range of motion ankle dorsiflexion sub-score; FMA-ROM PF—Fugl-Meyer Lower Extremity range of motion ankle plantar flexion sub-score; SPPB—Short Physical Performance Battery; SD—standard deviation.

**Table 2:** Difference in the normalized ankle dorsiflexion torque during MIVC trainings with and without UVF in stroke participants (mean ± SD, *n* = 33).

Contraction Phase	Without UVF	With UVF	Percentage Difference	*p*-Value
***Whole contraction***				
Normalized mean torque (Nm/kg)	0.16 ± 0.11	0.17 ± 0.10	26.7% ± 52.6%	0.081
Normalized peak torque (Nm/kg)	0.20 ± 0.13	0.21 ± 0.11	19.4% ± 37.5%	0.075
Coefficient of variation	35.52% ± 12.20%	33.45% ± 8.04%	−1.2% ± 18.1%	0.782
%MIVC_Tq_	66.91% ± 20.41%	77.00% ± 20.82%	26.0% ± 46.8%	**0.007 ^†^**
***Initial (first 1/3 of contraction)***				
Normalized mean torque (Nm/kg)	0.16 ± 0.11	0.16 ± 0.09	16.3% ± 43.3%	0.851
Normalized peak torque (Nm/kg)	0.19 ± 0.12	0.20 ± 0.10	17.0% ± 37.0%	0.330
Coefficient of variation	30.80% ± 5.62%	33.13% ± 6.32%	8.7% ± 16.5%	**0.007 ^†^**
%MIVC_Tq_	69.86% ± 19.30%	75.24% ± 16.10%	16.3% ± 43.3%	0.155
***Mid (middle 1/3 of contraction)***				
Normalized mean torque (Nm/kg)	0.17 ± 0.12	0.19 ± 0.11	41.9% ± 103.7%	**0.006 ^†^**
Normalized peak torque (Nm/kg)	0.19 ± 0.13	0.21 ± 0.11	22.6% ± 39.9%	**0.044 ^†^**
Coefficient of variation	15.00% ± 23.58%	9.09% ± 8.02%	−5.9% ± 87.2%	**0.046 ^†^**
%MIVC_Tq_	72.23% ± 23.89%	87.89% ± 26.43%	41.9% ± 103.7%	**0.001 ^†^**
***End (last 1/3 of contraction)***				
Normalized mean torque (Nm/kg)	0.14 ± 0.10	0.15 ± 0.10	36.4% ± 74.0%	**0.022 ^†^**
Normalized peak torque (Nm/kg)	0.17 ± 0.12	0.19 ± 0.12	31.0% ± 55.8%	**0.006 ^†^**
Coefficient of variation	36.35% ± 13.73%	35.12% ± 8.82%	2.4% ± 24.4%	0.526
%MIVC_Tq_	58.56% ± 23.66%	68.09% ± 24.32%	36.4% ± 74.0%	0.004 *

**Note:** UVF—ultrasound-imaging-based visual feedback; MIVC—maximal isometric voluntary contraction; SD—standard Deviation. Significant differences are represented in **bold** format. *: significant difference based on paired-*t* test; †: significant difference based on Wilcoxon signed rank tests.

**Table 3:** Difference in the TA muscle thickness during MIVC trainings with and without UVF in stroke participants (mean ± SD, *n* = 33).

Contraction Phase	Without UVF	With UVF	Percentage Difference	*p*-Value
***Whole contraction***				
Mean thickness (mm)	28.5 ± 2.4	28.6 ± 2.4	0.4% ± 0.0%	0.164
Peak thickness (mm)	28.8 ± 2.4	28.9 ± 2.4	0.5% ± 0.0%	0.145
Coefficient of variation	0.3% ± 0.4%	0.3% ± 0.4%	8.6% ± 0.6%	0.681
%MIVC_MT_	99.8% ± 2.4%	100.2% ± 2.3%	0.4% ± 0.0%	0.173
***Initial (first 1/3 of contraction)***				
Mean thickness (mm)	28.4 ± 2.4	28.5 ± 2.4	0.4% ± 0.0%	0.191
Peak thickness (mm)	28.6 ± 2.4	28.7 ± 2.4	0.4% ± 0.0%	0.187
Coefficient of variation	0.3% ± 0.4%	0.3% ± 0.3%	7.5% ± 0.5%	0.860
%MVC_MT_	99.8% ± 2.4%	100.2% ± 2.4%	0.4% ± 0.0%	0.187
***Mid (middle 1/3 of contraction)***				
Mean thickness (mm)	28.5 ± 2.4	28.7 ± 2.4	0.5% ± 0.0%	0.092
Peak thickness (mm)	28.6 ± 2.4	28.8 ± 2.4	0.6% ± 0.0%	0.045 *
Coefficient of variation	0.1% ± 0.2%	0.2% ± 0.2%	23.0% ± 0.8%	**0.044 ^†^**
%MIVC_MT_	99.8% ± 2.5%	100.3% ± 2.5%	0.5% ± 0.0%	0.095
***End (last 1/3 of contraction)***				
Mean thickness (mm)	28.5 ± 2.4	28.7 ± 2.5	0.6% ± 0.0%	0.099
Peak thickness (mm)	28.7 ± 2.5	28.8 ± 2.5	0.6% ± 0.0%	0.096
Coefficient of variation	0.2% ± 0.3%	0.2% ± 0.3%	18.9% ± 0.7%	0.788
%MIVC_MT_	99.7% ± 2.4%	100.2% ± 2.3%	0.6% ± 0.0%	0.112

**Note:** UVF—ultrasound-imaging-based visual feedback; MIVC—maximal voluntary contraction; SD—standard Deviation. Significant differences are represented in **bold** format. *: significant difference based on paired-*t* test; †: significant difference based on Wilcoxon signed rank tests.

**Table 4:** Correlation between the mean normalized ankle dorsiflexion toque and the mean absolute TA thickness during the MIVC trainings with and without UVF in stroke participants (*n* = 33).

Condition	Correlation Coefficient (r)	*p*-Value
***Combined two training conditions***		
Whole contraction	0.30	**0.016 ^†^**
Initial (first 1/3 of contraction)	0.37	**0.003 ^†^**
Mid (middle 1/3 of contraction)	0.30	**0.013 ^†^**
End (last 1/3 of contraction)	0.20	0.100
***Training with UVF only***		
Whole contraction	0.27	0.134
Initial (first 1/3 of contraction)	0.31	0.079
Mid (middle 1/3 of contraction)	0.28	0.111
End (last 1/3 of contraction)	0.21	0.242
***Training without UVF only***		
Whole contraction	0.32	0.072
Initial (first 1/3 of contraction)	0.36	**0.040 ^†^**
Mid (middle 1/3 of contraction)	0.34	0.051
End (last 1/3 of contraction)	0.20	0.271

**Note:** TA—tibialis anterior; UVF—ultrasound-imaging-based visual feedback. Significant differences are represented in **bold** format. †: significant correlation based on Spearman’s correlation test.

**Table 5:** Correlation between the peak normalized ankle dorsiflexion toque and the peak absolute TA muscle thickness during the MIVC trainings with and without UVF in stroke participants (*n* = 33).

Condition	Correlation Coefficient (r)	*p*-Value
***Combined two training conditions***		
Whole contraction	0.30	**0.014 ^†^**
Initial (first 1/3 of contraction)	0.35	**0.005 ^†^**
Mid (middle 1/3 of contraction)	0.31	**0.011 ^†^**
End (last 1/3 of contraction)	0.24	0.054
***Training with UVF only***		
Whole contraction	0.25	0.157
Initial (first 1/3 of contraction)	0.36	0.039 *
Mid (middle 1/3 of contraction)	0.25	0.155
End (last 1/3 of contraction)	0.23	0.206
***Training without UVF only***		
Whole contraction	0.34	0.054
Initial (first 1/3 of contraction)	0.35	**0.049 ^†^**
Mid (middle 1/3 of contraction)	0.36	**0.043 ^†^**
End (last 1/3 of contraction)	0.24	0.172

**Note:** TA—tibialis anterior; UVF—ultrasound-imaging-based visual feedback. Significant differences are represented in **bold** format. *: significant correlation based on Pearson’s correlation test; †: significant correlation based on Spearman’s correlation test.

## Data Availability

All data generated or analyzed during this study are included in this published article.

## Supplementary Materials

The following supporting information can be downloaded at: https://www.mdpi.com/article/10.3390/bios15060365/s1; Table S1: CONSORT checklist of information to include when reporting randomized crossover trials; Table S2: Reliability tests for the wearable SMG system in muscle thickness measurement; Table S3: Reliability test for visual feedback target identification.

## Abbreviations

The following abbreviations are used in this manuscript:

CONSORT	Consolidated Standards of Reporting Trials
EEG	Electroencephalogram
FIM	Functional Independence Measure
FMA-LE	Fugl-Meyer Assessment for Lower Extremity
fNIRS	Functional near-infrared spectroscopy
ICTRP	International Clinical Trials Registry Platform
MIVC	Maximal isometric voluntary contraction
MIVCMT	MIVC muscle thickness
MIVCTq	MIVC torque
PSSUQ	Post-Study System Usability Questionnaire
ROM	Range of motion
SMG	Sonomyography
SPPB	Short Physical Performance Battery
SUS	System Usability Scale
TA	Tibialis anterior
UVF	Ultrasound-imaging-based visual feedback
